# Complex interplay of science reasoning and vaccine hesitancy among parents in Shanghai, China

**DOI:** 10.1186/s12889-024-17990-4

**Published:** 2024-02-23

**Authors:** Felicia Zhang, Jia Ren, Colin Garon, Zhuoying Huang, John Kubale, Abram L. Wagner

**Affiliations:** 1 Department of Epidemiology, University of Michigan, 48109 Ann Arbor, MI USA; 2 Department of Immunization Program, Shanghai Municipal Centers for Disease Control & Prevention, 200336 Shanghai, China; 3 Department of Anthropology, University of Michigan, 48109 Ann Arbor, MI USA; 4 Institute for Social Research, University of Michigan, 48109 Ann Arbor, MI USA

**Keywords:** Science literacy, Papillomavirus vaccines, Vaccination, China, Surveys and questionnaires

## Abstract

The psychosocial underpinnings of vaccine hesitancy are complex. Research is needed to pinpoint the exact reasons why people hesitate to vaccinate themselves or their children against vaccine-preventable diseases. One possible reason are concerns that arise from a misunderstanding of vaccine science. We examined the impact of scientific reasoning on vaccine hesitancy and human papillomavirus (HPV) vaccination intent through a cross-sectional study of parents of vaccine-eligible children (*N* = 399) at immunization clinics in Shanghai, China. We assessed the relationship between science reasoning and both vaccine hesitancy and HPV vaccine acceptance using general additive models. We found a significant association between scientific reasoning and education level, with those with less than a high school education having a significantly lower scientific reasoning that those with a college education (ß = -1.31, p-value = 0.002). However, there was little evidence of a relationship between scientific reasoning and vaccine hesitancy. Scientific reasoning therefore appears not to exert primary influence on the formation of vaccine attitudes among the respondents surveyed. We suggest that research on vaccine hesitancy continues working to identify the styles of reasoning parents engage in when determining whether or not to vaccinate their children. This research could inform the development and implementation of tailored vaccination campaigns.

## Introduction

Addressing vaccine hesitancy within a specific population requires an understanding of how this hesitancy arises in the first place. Vaccine hesitancy and the refusal of vaccines despite their availability in an area could blunt the impact of the introduction of new vaccines, like human papillomavirus (HPV) vaccine, which is theoretically highly cost effective even in low and middle-income countries [1]. Vaccine hesitancy has also been linked to outbreaks of diseases, like measles, that have long been vaccine-preventable [2, 3]. Efforts to control the spread of other vaccine-preventable diseases, including COVID-19, are also stymied by widespread vaccine hesitancy [4]. The WHO’s designation of vaccine hesitancy as a top threat to global health in 2019 [3], and its earlier efforts to study vaccine hesitancy in the early 2010s [5], therefore, have proven prescient.

Studying vaccine hesitancy is difficult because it has many potential psychosocial causes [6]. Some commonly cited factors include lack of information, anxieties about vaccine safety, distrust in government, and desire for autonomy, all of which interect with structural barriers, such as concerns about affordability, and inconvenience of access [7]. Landmark studies of vaccine hesitancy focus on Europe and North America [8, 9], limiting the generalizability of our understanding to other settings, particularly low- and middle-income countries (LMICs). In the last two decades, the WHO has greatly increased the number of vaccines recommended to be included in the Expanded Program on Immunization worldwide [10]; their roll-out could be imperiled if vaccine hesitancy remains understudied in diverse global contexts.

Careful study of vaccine hesitancy requires development of scales. One such scale, the Parent Attitudes about Childhood Vaccines (PACV) assesses vaccine hesitancy directly through a series of questions aimed at assessing parents’ preferences for their child’s vaccination. The PACV correlates with other measures of vaccine hesitancy, like Gust et al.’s categorization of individuals into ‘Immunization Advocates’, ‘Go along to get along’, ‘Health Advocates’, ‘Fencesitters’, and ‘Worried’ [11, 12].

One determinant of vaccine hesitancy could be scientific reasonsing [13]. Scientific reasoning differs from but relates to scientific literacy, assessing respondents’ ability to understand scientific methods and replicate them in their own thinking. Instead of measuring content knowledge, scientific reasoning focuses on cognitive abilities and critical thinking that lets someone respond to new or unforeseen situations [14]. Understand how, or if, vaccine hesitancy correlates with vaccine hesitancy could help inform the development of educational interventions. We measured scientific reasoning using the Scientific Reasoning Scale (SRS), developed and validated by Drummond and Fischhoff [15]. A study undertaken during the development of the scale found that people with higher SRS scores tend to agree with scientific consensus, including on the safety of vaccines, though scientific reasoning was unrelated to political liberalism or religiosity [15]. The SRS has been used to study laypeople’s adherence to beliefs and practices unsupported by scientific consensus, including alternative medicine [16], and has been translated for use in Türkiye [17].

There is a relative paucity of vaccine hesitancy information outside of Europe and North America. Accordingly, in a cross-sectional study of parents in Shanghai, China, we aimed to (1) examine demographic differences in scientific reasoning; (2) characterize the relationship between scientific reasoning and vaccine hesitancy, including the subdomains described in PACV; and (3) assess the relationship between scientific reasoning and HPV vaccine acceptance. By doing so, we may query not only whether interventions aimed at developing laypeople’s scientific reasoning skills might increase vaccine acceptance, but also the extent to which people use scientific reasoning in their intentions and decisions surrounding adolescent vaccination. Overall, this study investigates the relationship between scientific reasoning and vaccine attitudes in an upper-middle-income country through a cross-sectional study of parents with vaccine-age children in Shanghai, China.

## Materials and methods

### Study population

Data for this study were collected between May and July 2019, in Shanghai, China. Investigators sampled 40 townships randomly (out of all districts in Shanghai except Chongming, an island district which is among the furthest away from downtown Shanghai), based on the size of their population according to the 2010 Census. Parents at immunization clinics were sampled within each township. The immunization clinics typically service children < 5 years old and < 2 years old. The eligibility criterion for the parents was having a child of ≤ 18 years old. Parents with more than one child could be included as long as one of the children was aged ≤ 18 years.

### Study design

This is a cross-sectional survey with a convenience sampling scheme. Based on the multistage selection procedure, we generated probability of selection weights that were applied to the analysis to weight the respondents to be reflective of the general population of Shanghai. The study is a subset of a larger study of parents in Shanghai. In that study, parents were sampled from schools and clinics. In that study, 1,183 individuals were approached to participate; of these 66 refused and 76 did not complete the survey, yielding a final sample size of 1.041 (88%). This study comprises the 399 individuals sampled from clinics (and excluding those sampled from schools), because the clinic sample completed a longer survey including the full SRS.

For the larger study, 1,183 individuals in Shanghai were approached to participate in the survey. Of these, 66 refused to participate and 76 started but did not complete the survey, yielding a final_sample_size_of_1,041_(88.0%)._Subsequently,_20 grandparents who had been included were excluded, leading to a final_sample_size_of_1,021.

### Questionnaire

The questionnaire was given in simplified Chinese (Mandarin). Participants responded to written questions about their sociodemographic background, including age, sex, educational history, monthly family income, and child’s age. The participants also provided information about their residency. Residency was split into 3 categories: Shanghai locals, non-locals from other urban areas, and non-locals from rural areas.

### SRS11

Scientific reasoning was measured through an 11  -item scale (SRS11), taken from the Scientific Reasoning Scale (SRS) created by Drummond and Fischhoff. A native Chinese speaker translated the SRS11 into Mandarin and it was backtranslated by someone else. A series of eleven true/false questions were asked to participants, assessing their understanding of the scientific process. The variable SRS11 is a sum of how many questions participants answered correctly, from 0 questions answered correctly to 11 questions answered correctly.

### PACV100

One of the outcome variables from this study is vaccine hesitancy (PACV100), which comes from the 15-item Parental Attitudes towards Childhood Vaccines (PACV) scale. The scale was developed in English, and was translated into Mandarin Chinese. The original scale, validated through a U.S.-based survey, was divided into 3 domains: general attitudes, safety and efficacy, and vaccination behaviors. Responses were simplified into 3 categories: a point value of 0 for those most confident and least hesitant about vaccines, 2 for those responses associated with the most hesitancy about vaccines, and a score of 1 for responses that were in between. Cumulative scores were then rescaled to have a range between 0 and 100. In the original formulation of the scale in the U.S., items within a domain were summed and the sum dichotomized into those hesitant or not. For this study, instead of dichotomizing the variable, we kept it as a continuous outcome, which allowed us to examine more thoroughly the patterns of vaccine hesitancy beyond a dichotomous measure. The psychometric properties of PACV100 in China have previously been published [18].

### HPV Vaccine acceptance

We measured HPV vaccine acceptance for a hypothetical daughter and son through the following question: “Assume for the moment that the HPV vaccine is not mandatory but is free. If you had a [daughter| son], how willing would you be to give your [daughter| son] an HPV vaccine at 12 years of age?” Responses were on a 5-point scale: “not at all willing,” “not very willing,” “unsure,” “somewhat willing,” and “very willing.”

### Covariates

The covariates in this study included the parent’s relationship to the child (mother, father, or grandparent), education level of the parent (< high school, high school, vocational school, college, graduate school), residency status (urban non-locals, rural non-locals, and Shanghai locals), income (0–9,999, 10,000–19,999, and 20,000+), and age (20–29, 30–39, 40–49, 50+).

### Statistical analysis

We assumed, *a priori*, that SRS11 and PACV100 would be wkewed and not normally distributed. For bivariate statistics, we accordingly report the median and interquartile range as measures of spread.

We conducted three sets of multivariable regression models. All models include relationship to child (e.g., mother, father, grandparent), education, residency, income, and age as covariates based on *a priori* considerations of important confounders. In the first model, we examined the relationship between demographic variables and scientific reasoning using a linear regression model. We found this model held for assumptions of normality of residuals and homoscedasticity.

In the second model, we examined the relationship between scientific reasoning and vaccine hesitancy. Because the shape of the relationship between these variables is unclear, we modeled scientific reasoning using a spline term within a general additive model. In brief, this is a type of model where the predictor variable (i.e., scientific reasoning) is not assumed to have a monotonic or continuous relationship with the outcome (i.e., vaccine hesitancy). Instead the outcome is the sum of several functions with non-linear relationships [19] We visually interpret the relationship between scientific reasoning and vaccine hesitancy through an examination of a graph of the smoothing function.

In the third models, we examined HPV vaccine acceptance as the outcome, with scientific reasoning and vaccine hesitancy as predictors with spline terms. This model excludes grandparents (*n* = 5) due to small cell counts. Since both SRS11 and PACV100 are included as independent variables, the model outputs an estimate of their direct effect on the outcome.

All proportions and analyses were weighted based on the sampling scheme. We analyzed the data in R with package mgcv. Significance was assessed at an alpha level of 0.05.

## Results

The study population consisted of 399 individuals, with a median SRS11 score of 5 (IQR 4–7) and a median PACV100 (vaccine hesitancy score) of 33.33 (IQR:23.3–43.3) (Table 1). Most participants were mothers (82.1%), locals (56.5%), and between the ages of 30–39 (72.7%). The most common highly attained education level was college (48.0%), and the income levels of the participants were almost evenly distributed between 0 and 9,999 renminbi (RMB) (32.2%), 10,000–19,999 RMB (36.2%), and ≥ 20,000 RMB (31.7%), as seen in Table 1. Those with a higher level of education had a higher median SRS11. Vaccine hesitancy was relatively high among those with less education, among those who were rural non-local residents, and among those ≥ 50 years.

**Table 1:** Distribution of demographic variables, Shanghai, China, 2019.

		Count (col. %)	Median SRS11 (IQR)	Median PACV100 (IQR)
**Overall**		399	5 (4–7)	33.33 (23.3–43.3)
**Relationship to Child**	Mother	318 (82.1%)	5 (4–7)	33.3 (23.3–42.5)
	Father	76 (17.1%)	6 (4–7)	36.7 (26.7–46.7)
	Grandparent	5 (0.8%)	3 (1–6)	30.0 (26.7–53.3)
**Education**	< High school	37 (11.7%)	5 (3–6)	36.7 (30-46.7)
	High school	48 (12.6%)	5 (3–6)	33.3 (26.7–43.3)
	Vocational school	91 (22.3%)	5 (4–6)	36.7 (28.3–43.3)
	College	201 (48.0%)	6 (4–7)	30.0 (20.0–40.0)
	Graduate school	22 (4.7%)	7 (5.5-9)	33.3 (17.5–40.0)
**Residency Status**	Local	219 (56.5%)	5 (4–7)	33.3 (20.0-43.3)
	Urban non-local	72 (15.1%)	6 (4–7)	30.0 (20.0–40.0)
	Rural non-local	104 (28.4%)	5 (4–7)	36.7 (30.0-43.3)
**Income**	< 10,000 RMB	112 (32.2%)	5 (3–6)	33.3 (26.7–45.0)
	10,000–19,999 RMB	152 (36.2%)	5 (4–7)	33.3 (20.0-43.3)
	≥ 20,000 RMB	131 (31.7%)	6 (4–8)	30.0 (20.0-43.3)
**Age of parent**	20–29 years	23 (6.5%)	5 (3.5-7)	33.3 (26.7–45.0)
	30–39 years	295 (72.7%)	6 (4–7)	33.3 (23.3–43.3)
	40–49 years	75 (19.8%)	6 (5–7)	30.0 (16.7–40.0)
	≥ 50 years	5 (1.0%)	4 (4–4)	43.3 (33.3–43.3)

Note: IQR: interquartile range; RMB: renminbi

The linear regression of the SRS11 variable on sociodemographic variables is shown in Table 2. Controlled for the other variables, there was a positive relationship between level of education and SRS11 score. For example, those with less than a high school education had 1.31 points lower score than those of a college education.

**Table 2:** Linear regression of demographic characteristics on scientific reasoning, Shanghai, China, 2019

		Beta Estimate	SE	***P***-value
**Education**	≥ Graduate school	0.98	0.53	0.0654
	College	ref		
	Vocational	-0.58	0.29	0.0441
	High school	-0.74	0.46	0.1108
	< High school	-1.31	0.44	0.0032
**Relationship**	Grandparent	-1.86	3.68	0.6132
	Mother	0.35	0.30	0.2489
	Father	ref		
**Residency Status**	Local	ref		
	Urban non-local	0.28	0.33	0.3931
	Rural non-local	0.30	0.32	0.3596
**Income**	< 10,000 RMB	ref		
	10,000–19,999 RMB	0.27	0.31	0.3806
	≥ 20,000 RMB	0.73	0.34	0.0320
**Age**	20–29 years	ref		
	30–39 years	-0.06	0.48	0.9022
	40–49 years	-0.43	0.54	0.4264
	≥ 50 years	-1.26	1.18	0.2881

Note: RMB: renminbi; SE: Standard Error

**Fig. 1:**
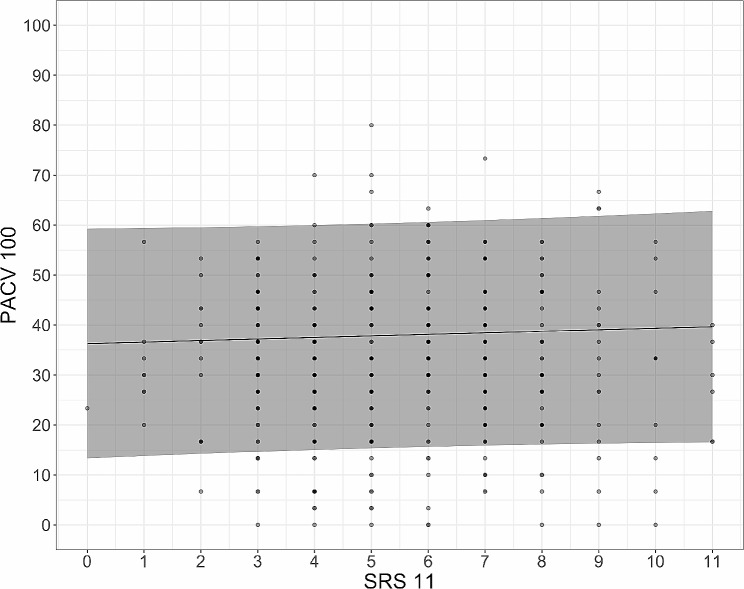
Relationship between the scientific reasoning scale (SRS11) and parent attitudes about childhood vaccines scale (PACV100) using a regression spline, Shanghai, China, 2019

Table 3 shows the model examining the association between vaccine hesitancy (PACV100) and SRS11, controlled for sociodemographic variables. This model does not show a significant relationship between SRS11 and PACV100 (graphically depicted in Fig. 1), neither was there a relationship between education and vaccine hesitancy. Vaccine hesitancy was relatively high among rural non-locals (4.92 points higher than locals, *P* = 0.0223).

**Fig. 2:**
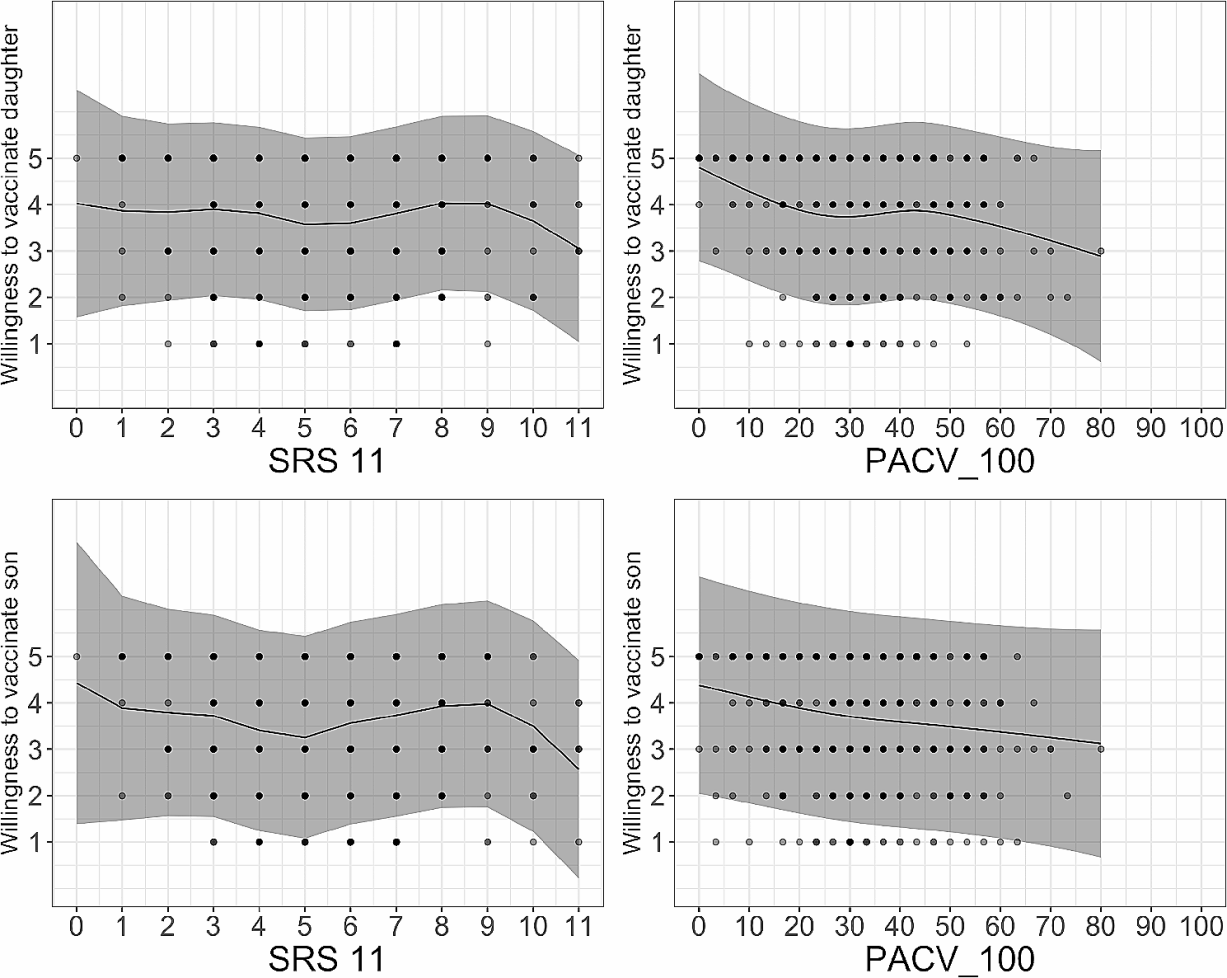
Relationship between the scientific reasoning scale (SRS11) or the parent attitudes about childhood vaccines scale (PACV100) and willingness to vaccinate a daughter (top row) or son (bottom row) using regression splines, Shanghai, China, 2019

Table 4 shows the models assessing the impact of vaccine hesitancy and scientific reasoning on acceptance of an HPV vaccine for a 12-year-old daughter or son. Graphically, there is little evidence for associations– perhaps a subtle, negative relationship between PACV100 and willingness to vaccinate either a son or a daughter (Fig. 2), whereas this relationship is less clear for SRS11. For vaccinating a daughter, willingness was higher among mothers, those in the highest income brackets, and in younger ages. Patterns were similar for vaccinating a son, except there was no significant difference by age or between mothers and fathers.

**Table 3:** General additive model of scientific reasoning on vaccine hesitancy, Shanghai, China, 2019

		Beta Estimate	SE	***P-***value
**SRS11**		NA^a^		0.3680^b^
**Education**	≥ Graduate school	1.84	3.55	0.6041
	College	ref		
	Vocational	2.21	1.93	0.2538
	High school	-2.08	3.08	0.4993
	< High school	1.68	2.98	0.5734
**Relationship**	Grandparent	18.71	24.53	0.4462
	Mother	-1.23	1.99	0.5382
	Father	ref		
**Residency Status**	Local	ref		
	Urban non-local	-1.52	2.18	0.4878
	Rural non-local	4.92	2.15	0.0223
**Income**	< 10,000 RMB	ref		
	10,000–19,999 RMB	-1.16	2.06	0.5754
	≥ 20,000RMB	-0.87	2.26	0.7016
**Age of parent**	20–29 years	ref		
	30–39 years	0.92	3.17	0.7712
	40–49 years	-3.33	3.62	0.3574
	≥ 50 years	-7.38	7.87	0.3486

Note: RMB: renminbi; SE: Standard Error

^a^ Relationship graphically depicted in Fig. 1

^b^ P-value of smooth term

^c^ Refer to Fig. 1 for graphical representation of model

**Table 4:** General additive model of HPV vaccine acceptance on scientific reasoning and vaccine hesitancy, Shanghai, China, 2019

		Willingness to vaccinate a daughter			Willingness to vaccinate a son		
		Beta Estimate	SE	***P***-value	Beta Estimate	SE	***P***-value
**SRS11**		NA^a^		< 0.0001^b^	NA^a^		< 0.0001^b^
**PACV100**		NA^a^		< 0.0001^b^	NA^a^		< 0.0001^b^
**Education**	≥ Graduate school	-0.29	0.32	0.3029	-0.13	0.34	0.7074
	College	Ref			Ref		
	Vocational	0.29	0.16	0.0672	0.07	0.18	0.7136
	High school	-0.20	0.25	0.4305	-0.45	0.30	0.1306
	< High school	0.25	0.24	0.3020	0.27	0.28	0.3422
**Relationship**	Mother	0.41	0.16	0.0111	0.31	0.19	0.1001
	Father	ref			ref		
**Residency Status**	Local	ref			ref		
	Urban non-local	0.11	0.18	0.5399	-0.25	0.21	0.2256
	Rural non-local	0.20	0.18	0.2601	0.24	0.21	0.2429
**Income**	< 10,000 RMB	ref			ref		
	10,000–19,999 RMB	0.13	0.17	0.4289	-0.30	0.20	0.1220
	≥ 20,000 RMB	0.4 1	0.18	0.0261	-0.05	0.21	0.8040
**Age of parent**	20–29 years	Ref			ref		
	30–39 years	-0.49	0.26	0.0618	-0.63	0.30	0.0351
	40–49 years	-0.72	0.29	0.0153	-0.75	0.34	0.0293
	≥ 50 years	-0.74	0.63	0.24	0.02	0.84	0.9840

Note: RMB: renminbi; SE: Standard Error

^a^ Relationship graphically depicted in Fig. 2

^b^ P-value of smooth term

^c^ Refer to Fig. 2 for graphical representation of model

## Discussion

The introduction of a new vaccine into a population requires population support, particularly in situations where there is not a mandate to obtain the vaccine. In China, which has licensed but has not mandated vaccination against HPV, we conducted a study to understand the correlations among scientific reasoning, vaccine hesitancy, and HPV vaccination intent among parents. Findings can help guide the development of vaccination promotion materials.

### Correlates of scientific reasoning

Our results indicate that education level significantly predicts scientific reasoning. Survey respondents who had not received college education had lower SRS scores than those with college-level or advanced education. These results are consistent with those of both the original SRS, which significantly correlated reported education level to higher SRS scores [15], and the Turkish SRS translated by Kaygisiz et al., who found that graduate students scored over a point higher than undergraduates on average [17]. It also accords with one study, which, using a different measure of scientific reasoning than the SRS, found that there was monotonically higher scientific reasoning among Chinese children and young adults following greater levels of education [20].

### Correlates of vaccine hesitancy

We did not find any significant correlations between scientific reasoning and vaccine hesitancy. These results ought to be evaluated against i social scientists’ broader search for cognitive factors driving laypeople’s belief in and adherence to the ‘unscientific’: conspiracy theories, pseudoscience, alternative medicine, and the anti-vaccination movement (e.g., [13]). In some cases, scientific reasoning proves salient. In their original article, Drummond and Fischhoff observe that the SRS predicted belief in the safety of vaccines and GMOs among US survey respondents, if not belief in the Big Bang or global warming [15], and the survey in Türkiye found negative correlations between respondents’ SRS scores and their self-reported beliefs in and use of complementary and alternative medicine (CAM) [16]. In other cases, scientific reasoning has appeared unrelated to the spread of ‘epistemically suspect beliefs’: for example, a recent survey exploring susceptibility to online health misinformation amongst American respondents discarded the SRS as a predictor [21].

One demographic correlate of vaccine hesitancy was residency– with rural non-local individuals (i.e., more recent migrants into Shanghai from rural areas) more likely to be hesitant. Given the on-going, large-scale migration of individuals into cities in China [22], understanding vaccine hesitancy in recent migrants could be key to mitigating outbreaks of vaccine-preventable diseases.

### Correlates of HPV vaccination intent

We found little evidence of an association between either SRS11 or PACV100 and HPV vaccination intent. The relationship between PACV100 and HPV vaccination intent has a stronger direction of association, but more evidence will be needed, likely in a larger sample size, to determine whether such an association exists.

In our outcome of HPV vaccination intent, age, income, and parental status (father vs. mother) were associated with greater intent of vaccinating a daughter. We note that many of these patterns varied similarly to the models with vaccine hesitancy as the outcome. Interestingly, we found mothers to have lower intent to vaccinate their children. In other contexts (e.g., COVID-19 vaccine intent in Qatar [23]) a reverse association has been observed, with mothers having greater intent. This difference could lie in various cultural factors, knowledge of different vaccine-preventable antigens, or other experiences.

### Use of scientific reasoning

A common conclusion, evident across the literature, is that scientific reasoning appears highly context-specific. The original SRS was developed as a corrective to ‘content-based’ measures of scientific literacy, seeking instead to identify modes of reasoning mobilized in experimental science that laypeople might apply across contexts. Studies of the SRS have since explored context-based reasoning in several ways:

#### Efforts to recontextualize or further decontextualize the SRS

Some scholars have responded to the SRS with concerns that its examples are too abstract for some laypeople to grasp, thus skewing survey results. One revision of the SRS, for example, recalibrates 11 of its items to an overarching everyday premise: “Amir is overweight and wants to find a research-proven method to lose weight” [24]. This approach suggests that scientific reasoning is entirely engaged with context: it is a process of “rationaliz[ing] between competing pieces of evidence” in order to arrive at reliable and actionable understanding. Meanwhile, a study by Bašnáková et al attempted to parse when people applied abstract principles to a given case, versus when they reasoned heuristically from concrete examples. This study compared Slovak adults’ responses to two versions of the SRS: one context-free and one containing concrete information about a particular research domain [25]. An entirely ‘context-free’ SRS, however, has proven elusive. Comparing responses to the construct validity item of the SRS, the researchers observe that respondents correctly rejected the proposition that a teacher covering several mathematical topics including algebra and geometry need only use a geometry test to measure students’ mathematical abilities, but incorrectly assumed that a teacher covering domain X, including subdomains A, B, C, and D, need only test the students on subdomain D. Here, it appears possible that another piece of context–namely, that A, B, C, and D often appear in sequential order–may itself have skewed respondents’ understanding. There are, in other words, contexts beyond contexts: heuristic reasoning may find all sorts of grounds.

#### Identifying contexts that appear to skew scientific reasoning

A vaccine acceptance instrument developed in the U.S. and validated by Sarathchandra et al. discovered that scientific reasoning increased vaccine acceptance among respondents that self-identified as liberal and decreased vaccine acceptance among respondents self-identifying as conservative [13]. The authors attribute these results to ‘motivated cognition’: respondents skilled at scientific reasoning manipulated their thinking in order to arrive at their desired outcome. Such an analytic move is especially apt for research in the United States, where the anti-vaccine movement combines partisan political rhetoric with spurious pseudoscientific claims about vaccines’ side effects. The Scientific Reasoning Scale might here measure not only an individual’s capacity to reason scientifically, but also their ability to manipulate biased reasoning into apparent ‘scientificity.’

#### Using the SRS as context in survey-based research

Following their development of the SRS, Drummond and Fischhoff have used the Scientific Reasoning Scale not so much to measure survey respondents’ cognitive capacities, but rather as a priming tool that activates the ‘mindware’ necessary to evaluate evidence scientifically [26]. In these cases, the SRS operates as its own sort of context: a cue to respondents that they ought to operationalize scientific reasoning in order to respond to a given set of prompts.

Our data on parents of vaccine-eligible children in Shanghai, China may be interpreted in light of these layers of context. In our study, the original Scientific Reasoning Scale survey was translated directly into Chinese. We might consider, for example, whether a version of the SRS more adapted to the context of child vaccination–for example, a series of questions that follow a mother and father researching the HPV vaccine for their 6-year-old child–might provide a clearer context in which parents might exercise their scientific reasoning, or alternatively prime respondents to answer the survey questions about willingness to vaccinate their child differently. More generally, however, we might consider whether HPV vaccination in urban China is a question of scientific reasoning in the first place.

Studies of vaccine acceptance in China have correlated vaccine hesitancy with vaccine manufacturing and management scandals [27]. Whereas in the United States, rhetoric associated with vaccine hesitancy has centered upon controversies over government mandates and vaccine science, requiring parents to evaluate competing forms of evidence from scientists, government officials, and public figures (e.g., [28]), the contours of vaccine hesitancy in China might differ from other countries based on different policies and the available supply of domestic vaccines. We note in the past in the US, there have been concerns about insufficient government regulation of vaccines: the Cutter incident of 1955, for example, shook public confidence in the newly developed Salk polio vaccine [29]. Vaccine quality scandals are well documented in the Chinese media [27]. If regulatory, rather than scientific or partisan, concerns predominate amongst urban Chinese parents, then the kind of reasoning that drives vaccine hesitancy might similarly depart from that of the United States. Here, risk perception might take front seat: Will my child run afoul of an improperly produced, packaged, or administered vaccine? In this case, another scale might produce a stronger correlation with the PACV than the scientific reasoning scale.

### Strengths and limitations

The strength of this analysis is that the data was collected from a community-based study, although the study population was limited to those present at immunization clinics, who may be more predisposed to be pro-vaccine. The limitations of this study are that it is cross-sectional, meaning that data was collected during a single moment of time (in this case, over a small period of time). We therefore lack sufficient information to assess any temporal dimension or mediation to the relationship between vaccine hesitancy and scientific reasoning. We also lack the collection of other, potentially relevant data, including whether the participant had advanced scientific training or worked in a scientific field.

## Conclusions

The broadest conclusion to draw from this study is that vaccine hesitancy is complex and context-specific, and the success or failure of various survey instruments helps us to determine precisely what kind of reasoning guardians in a specific place and time engage in when deciding whether to vaccinate their children. Research exploring how exactly parents reason about vaccination, furthermore, might inform interventions tailored to those empirically observed forms of reasoning.

## Data Availability

The dataset is available at: 10.6084/m9.figshare.21777974.
